# Clinical Characteristics and Treatment Strategies in a Cohort of Patients with Tularemia: A Retrospective Multicenter Analysis of 65 Cases in Germany

**DOI:** 10.3390/antibiotics14111169

**Published:** 2025-11-20

**Authors:** Benjamin Arnold, Henning Trawinski, Nils Kellner, Hans-Martin Orth, Daniela Tominski, Agata Mikolajewska, Katja Rothfuss, Gesa Grupe, Dominik Ruf, Friedrich Reichert, Daniela Jacob, Klaus Heuner, Kathrin Marx, Christoph Lübbert

**Affiliations:** 1 Department of Infectious Diseases and Tropical Medicine, Hospital St. Georg gGmbH, 04129 Leipzig, Germany; 2 Division of Infectious Diseases and Tropical Medicine, Department of Medicine I, Leipzig University Medical Center, 04103 Leipzig, Germany; 3 Department of Gastroenterology, Hepatology and Infectious Diseases, Medical Faculty and University Hospital Düsseldorf, 40225 Düsseldorf, Germany; 4 Infectious Diseases Unit, Auguste Viktoria Klinikum, 12157 Berlin, Germany; 5 Center for Biological Threats and Special Pathogens, Strategy and Incident Response, Clinical Management and Infection Control, Robert Koch Institute, 13353 Berlin, Germany; 6 Department of Gastroenterology, Hepatology and Endocrinology, Robert Bosch Hospital, 70376 Stuttgart, Germany; 7 Department of Infectious Diseases, Respiratory and Critical Care Medicine, Charité-Universitätsmedizin, 13353 Berlin, Germany; 8 Department of Hematology, Oncology, Immunology, Palliative Medicine, Infectious Diseases and Tropical Medicine, Klinikum München-Schwabing, 80804 Munich, Germany; 9 Department of Pediatrics, Olgahospital, Klinikum Stuttgart, 70174 Stuttgart, Germany; 10 National Reference Laboratory for *Francisella tularensis*, Center for Biological Threats and Special Pathogens, Highly Pathogenic Microorganisms (ZBS 2), Robert Koch Institute, 13353 Berlin, Germany; 11 Cellular Interactions of Bacterial Pathogens, Center for Biological Threats and Special Pathogens, Highly Pathogenic Microorganisms (ZBS 2), Robert Koch Institute, 13353 Berlin, Germany; 12 Hospital Pharmacy, Hospital St. Georg gGmbH, 04129 Leipzig, Germany; 13 Interdisciplinary Center for Infectious Diseases, Leipzig University Medical Center, 04103 Leipzig, Germany

**Keywords:** *Francisella tularensis*, tularaemia, lymphadenopathy, arthropod bite, treatment, antibiotics, outcome

## Abstract

**Background:** In recent years, there has been a significant increase in cases of tularemia, a rare zoonotic disease caused by *Francisella tularensis*, in Europe. **Methods:** To investigate the epidemiological, clinical, and therapeutic characteristics of tularemia patients in Germany, we performed a retrospective evaluation of tularemia cases treated between 2010 and 2025 at selected treatment centers of the Permanent Working Group of Competence and Treatment Centers for High Consequence Infectious Diseases (STAKOB) at the Robert Koch Institute. **Results:** A total of 65 patients (median age: 48.5 years; 66.2% male) were identified. Most common manifestation was ulceroglandular (70.7%), followed by oropharyngeal (13.8%), pulmonary (10.8%), oculoglandular (7.7%), typhoidal (4.6%), and meningitic (4.6%). Serological confirmation of the diagnosis was achieved in all patients (90.8% ELISA, 46.2% Western blot). PCR-based direct pathogen detection was successful in 26.2%. Bloodstream infection was detected in 4.6%. Median incubation period was 7 days (IQR: 4–10), with fever being the most common symptom in 96.9% and lymphadenopathy in 46.2%. Median time to recovery was 56 days (IQR: 37–80) in patients diagnosed and treated early (≤3 weeks after symptom onset), compared to 84 days (IQR: 66–182) in patients with late diagnosis (>3 weeks after symptom onset; *p* = 0.015). Empirical therapy with beta-lactam antibiotics was initiated in 49.2% of cases. Following suspicion of tularemia, 96.9% received recommended treatment with fluoroquinolones, tetracyclines, or aminoglycosides. **Conclusions:** Delayed diagnosis and inappropriate initial therapy can significantly prolong disease courses and increase morbidity. Early treatment with effective antibiotics, considering the intrinsic beta-lactam resistance of *Francisella tularensis*, leads to faster recovery.

## 1. Introduction

Human infections with *Francisella* (*F.*) *tularensis*, a pleomorphic, Gram-negative, highly infectious intracellular bacterium, are rare. However, over the past decade, Germany has experienced a steady increase in the number of annually reported cases [1]. *F. tularensis* is highly resistant to environmental influences, thus capable of surviving in cold and humid conditions for months [2]. *F. tularensis* comprises four main subspecies: *F. tularensis* ssp. *tularensis* (Type A), *F. tularensis* ssp. *holarctica* (Type B), *F. tularensis* ssp. *mediasiatica*, and *F. tularensis* ssp. *novicida* [3]. Of these, Type A and Type B are of main clinical relevance. Type A strains are only prevalent in North America, while Type B strains are widely distributed over the Northern Hemisphere, including Europe, Siberia, and Japan. Differentiation between subspecies is of clinical importance. For instance, Type A is associated with a particularly high case fatality rate (CFR) and is therefore classified as a potential biological weapon [4,5], especially in view of the high infectivity of *F. tularensis*: intradermal or inhalation inoculation of only 10–15 organisms is sufficient to cause a clinically relevant infection [2].

The main reservoirs of the pathogen are various small wild mammals such as hares, rabbits, mice, rats, and squirrels, which can have seropositivity rates of over 10% in Germany [3]. However, the pathogen is also found in the environment, including contaminated water and soil. Humans become infected through direct or indirect contact with infected animals, their organs or blood (e.g., during skinning), or excretions. Transmission may also occur through skin and mucous membrane contact, consumption of undercooked contaminated meat (e.g., hares), ingestion of contaminated surface water or food, or inhalation of infectious dust [2,3]. In endemic areas, vectors such as ticks and biting insects are also significant contributors to transmission. Human-to-human transmission has not yet been described. Particularly vulnerable groups include hunters, forestry workers, game meat processors, taxidermists, farmers, and laboratory staff [2,3].

After an incubation period of typically 3–5 days (range: 1–21 days), patients may develop a variety of localized or systemic symptoms, depending on the site of entry, the virulence of the pathogen strain, and the infectious dose [2]. Early symptoms are often non-specific such as fever, chills, and headache, often accompanied by painful lymphadenopathy. The further disease course depends on the site of infection and manifests in various forms: ulceroglandular, glandular, oculoglandular, oropharyngeal, pulmonary, or typhoidal (a septicemic form with an unknown route of infection). In Central and Northern Europe, the ulceroglandular form is the most common clinical presentation. It is characterized by an ulcerative skin lesion at the site of pathogen entry with regional lymphadenopathy. It usually develops after direct skin contact with infected animals or their tissues or following bites of infected ectoparasites (e.g., ticks, mosquitoes, horse flies, fleas). Morbidity and mortality depend on the *F. tularensis* subspecies involved: Type A (*F. tularensis* ssp. *tularensis*), particularly clade A1, is significantly more virulent than Type B (*F. tularensis* ssp. *holarctica*). Prior to the availability of effective antibiotic therapy, the CFR of Type A1 infections was 5% to 10%, with untreated pulmonary and typhoidal forms reaching up to 60% [4]. The CFR of Type B infections is significantly lower. To make matters worse, *F. tularensis* produces a class A beta-lactamase (FTU-1) that leads to resistance to all beta-lactam antibiotics, including carbapenems, which complicates empirical therapy [6,7].

The number of reported tularemia cases in Germany continues to rise, with a total of 214 cases reported in 2024 alone [8]. In Sweden, up to 1000 cases of tularemia are diagnosed annually, highlighting the geographical spread within Europe [9]. In view of this significant increase in case numbers, the aim of this study was to investigate the epidemiological and clinical characteristics as well as treatment approaches in a cohort of tularemia patients treated at selected infectious disease centers in Germany.

## 2. Results

### 2.1. Patients

Five of the seven STAKOB centers (Berlin, Düsseldorf, Leipzig, Munich, and Stuttgart) and their locally situated partners in associated infectious diseases contributed data for this analysis. Patients were either treated primarily or transferred to the centers for diagnostic and treatment. A total of 65 patients were evaluable, of whom 43 (66.2%) were male and 22 (33.8%) were female. Regarding age, the distribution was broad, ranging from 5–81 years, with a median age of 48.5 years. Demographic and selected disease course and outcome characteristics are shown in Table 1.

**Figure 1:**
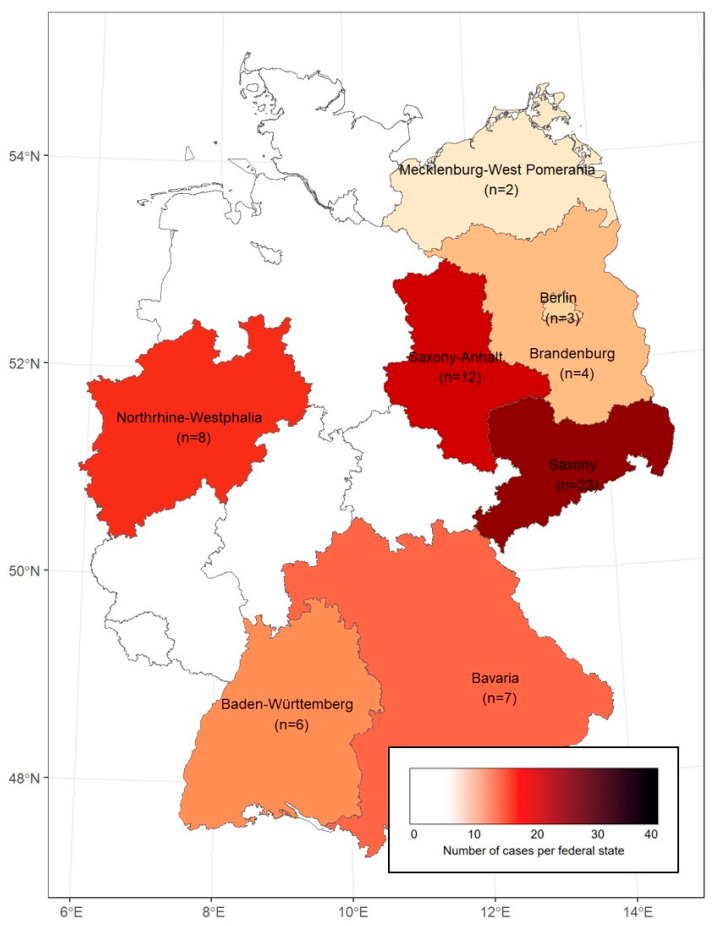
Geographic distribution of the study patients in Germany (federal states with analyzed cases are listed by name).

The geographical distribution of the study patients is shown in Figure 1, with the highest number of reported cases being located in Saxony and Saxony-Anhalt in Eastern Germany. The vast majority of cases were acquired domestically, while five cases were most likely imported from Sweden and one case from Portugal.

### 2.2. Clinical Courses

The median incubation period from potential pathogen exposure to symptom onset, which could be assessed in 43 patients, was 7 days (IQR: 4–10). Initial symptoms are shown in Table 2. The most common symptom was fever in 63/65 (96.9%) patients, followed by lymphadenopathy (46.2%).

**Figure 2:**
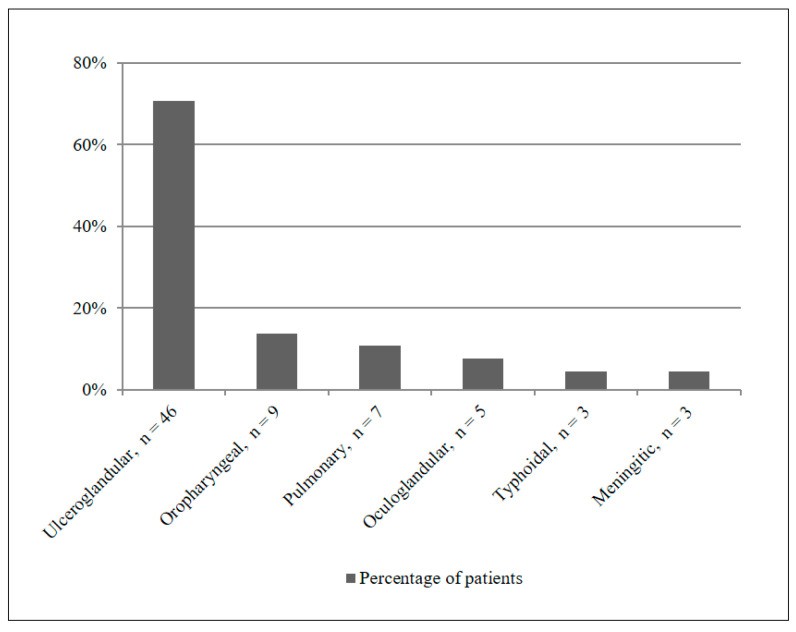
Distribution of tularemia manifestations in the study population.

The distribution of different clinical manifestations is shown in Figure 2. Ulceroglandular form was the most common presentation occurring in 46/65 (70.7%) patients (including two cases without clear ulceration), followed by oropharyngeal in 9/65 (13.8%), pulmonary in 7/65 (10.8%), oculoglandular in 5/65 (7.7%), typhoidal in 3/65 (4.6%), and meningitic in 3/65 (4.6%) patients. Overlaps between these clinical features were also observed, thus making it difficult to differentiate between them.

### 2.3. Routes of Transmission

The potential transmission routes are presented in Table 3. The mode of transmission could not be clarified in all cases since only 43/65 (66.2%) patients were able to provide evaluable information regarding potential exposure. However, tick bites were identified as most frequent, accounting for 14/65 (21.5%) cases, followed by hare contact in 8/65 (12.3%) cases, and mosquito bites and airborne transmission, each accounting for 5/65 (7.7%) cases.

### 2.4. Laboratory Findings

The median leucocyte count was 9.9 (IQR: 8.0–10.5) Gpt/L in the early diagnosis group and 8.8 (IQR: 7.3–10.3) Gpt/L in the late diagnosis group (*p* = 0.966). Median C-reactive protein (CRP) levels were 21.8 (IQR: 2.3–93.2) mg/L and 6.0 (IQR: 2.5–20.0) mg/L (*p* = 0.399), respectively. No statistically significant differences were observed for procalcitonin (PCT), interleukin-6 (IL-6), and lactate dehydrogenase (LDH) either.

Serological confirmation was obtained using ELISA IgM/IgG/IgA as a screening test in 21/24 (87.5%) patients in the early diagnosis group versus 38/41 (92.7%) in the late diagnosis group—in total, 59/65 (90.8%). Western blot as a confirmation test was positive in 9/24 (37.5%) versus 21/41 (51.2%)—in total, 30/65 (46.2%). Successful isolation of *F. tularensis* from blood cultures could be detected in 3/65 (4.6%) cases. Unfortunately, due to the retrospective nature of the data collection, it was not possible to determine whether blood cultures were taken from all patients and whether these were drawn before antibiotics were administered. PCR confirmed direct pathogen detection in 5/24 (20.8%) in the early diagnosis group versus 12/41 (29.3%) in the late diagnosis group—in total, 17/65 (26.2%). However, only 34/65 (47.6%) patients had evaluable PCR results.

Histopathological findings were available in 10/65 (15.4%) cases—3/24 (12.5%) in the early diagnosis group versus 7/41 (17.1%) in the late diagnosis group, showing characteristic granulomatous inflammation with presence of giant cells in tissue samples.

### 2.5. Risk Factors

The majority of patients (47/65; 72.3%) reported participation in outdoor activities or resided in rural areas. Ten patients (21.3%) were hunters, and 6 (12.8%) were part of an epidemic outbreak cluster, all of whom participated in hunting and skinning a hare that later turned out to be infected.

### 2.6. Comparison Between the Early and Late Diagnosis Groups

For comparison purposes, groups were classified as “early diagnosis” and “late diagnosis” based on a 3-week threshold. Of the 65 patients, 24 (36.9%) were in the early diagnosis group, and 41 (63.1%) in the late diagnosis group. The median time to diagnosis was 14 days (IQR: 10–20) in the early diagnosis group compared to 44 days (IQR: 28–56) in the late diagnosis group (*p* < 0.001) (Table 1).

Regarding the proportion of patients hospitalized and the duration of hospitalization, no difference was observed between both groups—15/24 (62.5%) were hospitalized in the early diagnosis group (median length of stay: 7 days [IQR: 5–16]) compared to 26/41 (63.4%) in the late diagnosis group (median length of stay: 11 days [IQR: 7–15]) (*p* = 0.470). In the early diagnosis group, 4/24 (16.7%) patients were suspected of having tularemia, and 19/24 (79.2%) were perceived to be afflicted by any other infectious agent. In contrast, 3/41 (7.3%) patients were suspected of suffering from tularemia or any other infectious agent in the late diagnosis group, with 6/41 (14.6%) initially suspected of having a malignant disease.

### 2.7. Antibiotic Treatment

A comprehensive overview of the data on antibiotic treatment, including the duration of therapy, is provided in Table 4. Thirty-two of 65 (49.2%) patients received empirical beta-lactam antibiotic treatment, with similar proportions in the early (11/24; 45.8%) and late (21/41; 51.2%) diagnosis groups. Sixty-three of 65 (96.9%) patients received antibiotics considered to be highly effective against *F. tularensis* from the beginning or sequentially: fluoroquinolones (mainly ciprofloxacin), tetracyclines (doxycycline), or aminoglycosides (almost exclusively gentamicin).

### 2.8. Surgical Interventions and Outcomes

Surgical procedures (mainly cervical and axillary lymphadenectomies as well as minor abscess incisions) were required in 9/24 (37.5%) patients in the early diagnosis group versus 29/41 (70.7%) in the late diagnosis group (*p* = 0.018), accounting for 38 patients (58.5%) in total (Table 1). All patients fully recovered from the disease. However, clinical relapse occurred in 8/24 (33.3%) patients in the early diagnosis group versus 23/41 (56.1%) in the late diagnosis group (*p* = 0.130), resulting in further surgical procedures necessary in 1/24 (4.2%) in the early diagnosis group compared to 4/41 (9.8%) in the late diagnosis group (*p* = 0.738).

The median time to full recovery was significantly longer in patients with delayed diagnosis—56 days (IQR: 37–80) in the early diagnosis group compared to 84 days (IQR: 66–182) in the late diagnosis group (*p* = 0.015).

## 3. Discussion

In this study, conducted within five of the seven treatment centers of the STAKOB network (covering seven of 16 federal states), we obtained data from 65 tularemia patients in Germany who were treated between 2010 and 2025 at selected centers for infectious diseases. However, during the same period, almost 1000 tularemia cases were mandatorily reported in Germany—mainly from the federal states of Bavaria and Baden-Wuerttemberg [1]. One possible explanation for this discrepancy is that many (>90%) tularemia patients were treated outside of treatment centers that collaborate in the STAKOB network. Among the 65 patients analyzed, we observed a geographical concentration in the Eastern federal states of Saxony and Saxony-Anhalt, accounting for more than half of the reported cases. This may reflect a selection bias, especially when taking into account that some cases were travel-associated or referred from other hospitals. On the other hand, regional differences in disease awareness, or actual differences in disease incidence across regions may play a role, although it should be noted that tularemia cases are reported from all federal states in Germany [8,10]. Interestingly, other studies have also reported significant differences in their nationwide distribution, for example, in the United States and Sweden, where various environmental factors play a major role [9,11].

In the present study—with the limitation that for 33.8% of patients no data or no memory of risk exposure and transmission routes were available—tick bites (21.5%) were the most frequently suspected transmission route, matching with the clinical presentation (ulceroglandular form as the most common presentation) and supporting the association between outdoor activities or living in the countryside and increased risk of infection in our cohort. A recent study by Nothdurfter et al. on tularemia cases in Baden-Wuerttemberg, Germany, over the past 10 years also concluded that approximately 20% of cases were transmitted by ticks [12], as already demonstrated by previous case reports from other regions in Germany [13]. Reliable data on the means of transmission are limited. Nevertheless, several studies have confirmed the presence of *F. tularensis* in ticks by means of PCR [14]. Recent studies from Central and Northern Europe point towards increased transmission of *F. tularensis* by ticks [9,15]. In contrast, in other geographical areas, different routes of transmission and clinical courses are more prominent. For instance, Erdem et al. reported that in a large case series in Turkey, 85.3% of patients presented with oropharyngeal tularemia [16], with contaminated water identified as the most common source of infection [16,17].

Furthermore, the median incubation period was 7 days in our study, which was slightly longer than reported in the literature [5]. Most patients presented with non-specific symptoms such as fever, night sweats, and fatigue. Lymphadenopathy was the second most common symptom (46.2%). In fact, studies from other countries have reported even higher lymphadenopathy rates—for instance, 95.5% in Turkey [16] and 49.6% in the United States [18]. Ulceroglandular/glandular manifestations were most common in our study (70.7%), exceeding rates reported in other recent publications (65.7% [19] and 58.1% [18]). With regard to surgical interventions (mainly cervical and axillary lymphadenectomies as well as minor abscess incisions), the rate differed significantly between the group with early diagnosis (37.5%) and the group with late diagnosis (70.7%; *p* = 0.018) in our study, underscoring the observation that surgical treatment is more frequently required when antibiotic treatment fails [5,18,20]. There were no deaths in our cohort, which can be attributed to the small number of patients and the generally low CFR for the subspecies prevalent in Europe (Type B)—*F. tularensis* ssp. *holarctica* [9,21]. Only 18.9% of the patients were clinically suspected to suffer from tularemia at primary admission, which explains the high proportion of empirical beta-lactam therapies (49.2%). In the early diagnosis group, nearly all patients were perceived to suffer from an infectious disease. On the other hand, the higher lymphadenopathy rate in the late diagnosis group was misleading and contributed to 14.6% being initially misdiagnosed with a suspected malignancy. The higher CRP levels observed in the early diagnosis group at admission were more indicative of inflammatory processes than in the late diagnosis group. However, the literature also describes cases in which CRP levels remain within the reference range during an infection [22].

The majority of our study patients were diagnosed through positive ELISA results. Bloodstream infection with confirmed positive blood culture was observed in only 4.6% of patients, which is in contrast to recent publications that observed an increasing incidence of *F. tularensis* bloodstream infections of up to 50% [20], possibly because tularemia was not initially suspected in many patients and the samples were therefore cultured for a too short period of time and no special culture media were used. The low number of positive findings from blood cultures contrasts with the diagnoses made by the National Reference Laboratory for *F. tularensis* at the Robert Koch Institute in Germany. Since 2021, approximately 80% of isolates to be confirmed there have come from blood cultures, accounting for approximately 14% to 22% of human cases reported in each year (unpublished data). This significant difference can possibly be explained, at least in part, by the referral behavior of the primarily commissioned peripheral laboratories to the specialized reference laboratory. In addition, our retrospective multicenter analysis covers the period from 2010 to 2025, while the aforementioned communication from the reference laboratory on blood cultures applies retroactively for 2021. The composition of commercially available blood culture media was also significantly improved between 2010 and 2020 with regard to the cultivation of *F. tularensis*. However, the successful cultivation of *F. tularensis* requires an incubation period of at least 5 days (up to 14 days) for blood cultures, which is not yet established in all peripheral laboratories. In terms of classic histopathological findings, granulomatous inflammation is typical for this infectious agent. However, these histopathological findings sometimes lead to misinterpretation and even inappropriate antituberculous treatment [16].

In our cohort, nearly half of the patients (49.2%) received initially one or more antimicrobial therapies based on beta-lactams, despite their known ineffectiveness regarding tularemia (presumably with the rationale of an empirical therapy of lymphadenitis including staphylococci and streptococci) [6,18,20]. The low incidence of tularemia in Germany may explain the lack of awareness of the disease among clinicians. In clinical practice, this means that many primary care physicians only initiated effective treatment with fluoroquinolones or doxycycline in a second step (usually after consulting a specialist) once the full course of beta-lactam therapy had been completed and no clinical improvement had been observed or the serological results for tularemia were available. Thus, delays in diagnosis and initiation of effective antimicrobial therapy contributed to significantly longer time to recovery—56 days in the early diagnosis group compared to 84 days in the late diagnosis group (*p* = 0.015). Large-scale studies have shown significantly better results with the use of highly effective antimicrobial agents [18,20]. In our cohort, only 26.2% received aminoglycosides. Significantly better results compared to other agents were observed for fluoroquinolones in terms of mean treatment duration—13 and 21 days (*p* = 0.025) in the early and late diagnosis groups, respectively. The mean treatment duration was also significantly longer for doxycycline in the late diagnosis group—18 versus 23 days (*p* = 0.042). The explanation for this is probably the higher disease burden in the late diagnosis group, underlined by the significantly higher need for surgical intervention (37.5% in the early diagnosis group compared to 70.7% in the late diagnosis group, *p* = 0.018) and the higher relapse rate (33.3% compared to 56.1%, *p* = 0.130). Other studies documented even higher clinical relapse rates of up to 86.1%, but clinical courses were particularly different [16].

The small number of cases limited our results and statistical analysis. Nevertheless, to our knowledge, this study represents the largest published cohort of tularemia cases in Germany to date. A recent analysis of 1163 cases of tularemia in the United States reported to the CDC showed positive clinical outcomes with first-line treatment with ciprofloxacin (adjusted odds ratio [aOR] for survival 5.3; 95% confidence interval [CI] 1.7–16.4), followed by doxycycline (aOR 4.9; 95% CI 1.9–12.6), and gentamicin (aOR 3.9; 95% CI 1.05–14.7) [18]. As already mentioned in another recent presentation of 14 tularemia cases from Germany [15], initial misdiagnoses were common in our cohort, leading to delayed diagnosis and multiple ineffective empirical antibiotic treatments. Ultimately, despite delays, and sometimes only after confirmation of diagnosis or after referral of the patients to our centers, 96.9% received recommended definitive treatment [23] with fluoroquinolones, doxycycline, or aminoglycosides.

## 4. Materials and Methods

### 4.1. Study Population and Definitions

Standardized retrospective data of tularemia patients treated between 2010 and 2025 were collected from the treatment centers of the Permanent Working Group of Competence and Treatment Centers for High Consequence Infectious Diseases (STAKOB). The data were shared in a data template using pseudonyms.

Potential cases of tularemia were detected by identifying all patients treated as inpatients and outpatients with a diagnosis coded under the International Classification of Diseases (revision 10, ICD-10) for tularemia (A21.0–A21.9). After assessing the data, only those cases meeting both clinical and laboratory criteria of tularemia were included in the final analysis. Laboratory confirmation was defined by either positive serology using enzyme-linked immunosorbent assays (ELISA) and Western blot and/or detection of *F. tularensis*-DNA using polymerase chain reaction (PCR) methods and/or successful cultural growth of *F. tularensis* from patient samples.

The timing of diagnosis was categorized as either “early” or “late” depending on how fast the patients obtained the specific diagnosis of tularemia—either ≤3 weeks or >3 weeks after the onset of symptoms. Early diagnosis within 3 weeks is challenging and involves direct detection of the pathogen, as serological tests often take 2–3 weeks to become positive after the onset of symptoms [2,5].

Full recovery was defined as a composite of normalization of body temperature, resolution of local findings, normalization of inflammatory parameters, and restoration of general condition.

### 4.2. Statistical Analysis

Statistical analysis was performed using R statistical software (version 4.5.1, https://www.r-project.org/). Continuous data are presented as mean with standard deviation (SD) or median with interquartile range (IQR), categorical data as numbers and percentages. Quantitative variables were compared using the Mann–Whitney U test, qualitative variables were compared using the Chi-square test or Fisher’s exact test, as appropriate. A *p*-value < 0.05 was considered statistically significant.

### 4.3. Ethics Approval

The study was conducted in accordance with the ethical guidelines of the 1964 Declaration of Helsinki and its later amendments and was approved by the local ethics committee (Saxonian Board of Physicians, Dresden, Germany, registration number EK-BR-54/21-1).

## 5. Conclusions

The diverse clinical presentations of tularemia, ranging from classic ulceroglandular forms to more severe and atypical manifestations, highlight the diagnostic and clinical complexity of the disease, which remains unfamiliar to many physicians in Germany. The classic risk group, which includes hunters, for example, can be expanded to include people who spend a lot of time outdoors during the summer months and become infected through arthropod bites (mainly ticks in Germany) and inhalation of dust contaminated with the pathogen. Increased awareness and early clinical suspicion are therefore essential, especially in endemic regions or in patients with a history of environmental exposure. Early diagnosis, including blood culture collection and subsequent treatment with recommended antibiotics, taking into account the intrinsic beta-lactam resistance of *F. tularensis*, leads to faster recovery and a reduced need for surgical intervention.

## Figures and Tables

**Table 1:** Demographic and selected disease course and outcome characteristics of the study patients.

Parameter	Overall (*n* = 65)	Early * Diagnosis (*n* = 24)	Late * Diagnosis (*n* = 41)	*p* Value
Male (%)	43 (66.2)	19 (79.2)	24 (58.5)	0.154
Female (%)	22 (33.8)	8 (21.1)	14 (51.9)	
Median age (years) (IQR)	48.5 (36–60)	49 (37–65)	47 (35–56)	0.272
Median time to diagnosis (days) (IQR)	28 (16–49)	14 (10–20)	44 (28–56)	
Hospitalization (%)	41 (63.1)	15 (62.5)	26 (63.4)	1.000
Median length of hospital stay (days) (IQR)	10 (5–15)	7 (5–16)	11 (7–15)	0.470
Surgical intervention (%)	38 (58.5)	9 (37.5)	29 (70.7)	0.018
Relapse (%)	31 (47.7)	8 (33.3)	23 (56.1)	0.130
Repeated surgery (%)	5 (7.7)	1 (4.2)	4 (9.8)	0.738
Median time to recovery (days) (IQR)	84 (45–112)	56 (37–80)	84 (66–182)	0.015

IQR = interquartile range. * The cut-off for “early” was ≤3 weeks and for “late” was >3 weeks after the onset of symptoms.

**Table 2:** Initial symptoms and frequency of occurrence in the study population.

Initial Symptoms	Overall (*n* = 65)	Early * Diagnosis (*n* = 24)	Late * Diagnosis (*n* = 41)	*p* Value
Fever (%)	63 (96.9)	22 (91.7)	41 (100.0)	0.257
Lymphadenopathy (%)	30 (46.2)	8 (33.3)	22 (53.7)	0.184
Night sweats (%)	23 (35.4)	8 (33.3)	15 (36.6)	1.000
Fatigue (%)	14 (21.5)	7 (29.2)	7 (17.1)	0.405
Loss of weight (%)	12 (18.5)	5 (20.8)	7 (17.1)	0.963
Headache (%)	10 (15.4)	3 (12.5)	7 (17.1)	0.891
Cough (%)	9 (13.8)	3 (12.5)	6 (14.6)	1.000
Myalgia (%)	3 (4.6)	1 (4.2)	2 (4.9)	1.000
Gastrointestinal symptoms (%)	1 (1.5)	0 (0.0)	1 (2.4)	1.000

* The cut-off for “early” was ≤3 weeks and for “late” was >3 weeks after the onset of symptoms.

**Table 3:** Potential transmission routes of *Francisella tularensis* in the study population.

Potential Route of Transmission	Overall (*n* = 65)	Early * Diagnosis (*n* = 24)	Late * Diagnosis (*n* = 41)	*p* Value
Tick bite (%)	14 (21.5)	8 (33.3)	6 (14.6)	0.145
Hare contact (%)	8 (12.3)	4 (16.7)	4 (9.8)	0.669
Airborne (%)	5 (7.7)	2 (8.3)	3 (7.3)	1.000
Mosquito bite (%)	5 (7.7)	2 (8.3)	3 (7.3)	1.000
Wild boar contact (%)	4 (6.2)	1 (4.2)	3 (7.3)	1.000
Rodent contact (%)	3 (4.6)	1 (4.2)	2 (4.9)	1.000
Domestic dog contact (%)	2 (3.1)	0 (0.0)	2 (4.9)	0.723
Deer contact (%)	1 (1.5)	0 (0.0)	1 (2.4)	1.000
Waterborne (%)	1 (1.5)	1 (4.2)	0 (0.0)	0.785
Unknown (%)	22 (33.8)	5 (20.8)	17 (41.5)	0.154

* The cut-off for “early” was ≤3 weeks and for “late” was >3 weeks after the onset of symptoms.

**Table 4:** Antibiotic treatment characteristics.

Antimicrobial Agents	Overall (*n* = 65)	Early * Diagnosis (*n* = 24)	Late * Diagnosis (*n* = 41)	*p* Value
Median duration of antimicrobial treatment (days) (IQR)	28 (21–35)	24 (21–28)	28 (21–37)	0.227
Fluoroquinolones (%)	43 (66.2)	16 (66.7)	27 (65.9)	1.000
Median duration of treatment with fluoroquinolones (days) (IQR)	14 (14–24)	14 (7–16)	14 (14–28)	0.025
Doxycycline (%)	46 (70.8)	18 (75.0)	28 (68.3)	0.771
Median duration of treatment with doxycycline (days) (IQR)	21 (14–21)	15 (14–21)	21 (21–28)	0.030
Aminoglycosides (%)	17 (26.2)	6 (25.0)	11 (26.8)	1.000
Median duration of treatment with aminoglycosides (days) (IQR)	10 (5–14)	5 (5–12)	10 (8–14)	0.330
Number of Antimicrobial Agents Given (Combination or Sequential)	Overall	1 Agent	2 Agents	3 Agents
	63	33	17	13
Fluoroquinolones	43	15	15	13
Doxycycline	46	18	15	13
Aminoglycosides	17	0	4	13

IQR = interquartile range. * The cut-off for “early” was ≤3 weeks and for “late” was >3 weeks after the onset of symptoms.

## Data Availability

Anonymized data will be available upon request. Contact benjamin.arnold@sanktgeorg.de.

## Abbreviations

The following abbreviations are used in this manuscript:

aOR	Adjusted odds ratio
CDC	Centers for Disease Control and Prevention
CFR	Case fatality rate
CI	Confidence interval
CRP	C-reactive protein
DNA	Deoxyribonucleic acid
ELISA	Enzyme-linked immunosorbent assay
ICD-10	International Classification of Diseases, revision 10
IL-6	Interleukin-6
IQR	Interquartile range
LDH	Lactate dehydrogenase
PCR	Polymerase chain reaction
PCT	Procalcitonin
RKI	Robert Koch Institute
SD	Standard deviation
STAKOB	Permanent Working Group of Competence and Treatment Centers for High Consequence Infectious Diseases at the Robert Koch Institute
